# Cost-effectiveness study of a home-based mental health hospitalization program for children and adolescents: study protocol

**DOI:** 10.1192/j.eurpsy.2025.1130

**Published:** 2025-08-26

**Authors:** I. Garcia Del Castillo, L. Heredia Castro, L. Caraballo Romero, A. Ortiz Villalobos, N. Fernandez Gomez, C. Cañadas, N. García, J. Andreo-Jover, T. Alonso, A. Gaona, A. Moreno Perez, M. F. Bravo Ortiz

**Affiliations:** 1 Psychiatry, La Paz University Hospital, Madrid; 2 Psychiatry, Principe de Asturias University Hospital, Alcala de Henares; 3 Psychiatry, Principe de Asturias University Hospital; 4 Psychiatry, Universidad Autonoma de Madrid; 5 Psychiatry, Hospital La Paz Institute for Health Research, Madrid, Spain

## Abstract

**Introduction:**

Psychiatric hospitalization to manage acute symptoms of mental disorders in children and adolescents may be necessary, although there is a growing concern about the clinical and economic effectiveness of psychiatric admission in this population. This suggests the need for the implementation of new intensive family and community care models that would reduce hospitalization.

Home hospitalization provides an opportunity to offer the child or adolescent with intensive treatment within the infrastructure of their home and the supervision of their family or primary caregivers. It therefore requires fewer resources than traditional inpatient treatment while allowing the patient‘s immediate environment to be involved in the treatment, potentially reducing the risk of a failed transition to the home once the patient is discharged.

**Objectives:**

The main objective is to analyze the cost-effectiveness of two Mental Health Home Hospitalization interventions for adolescents as an alternative to traditional hospitalization in two hospitals in Madrid, Spain (La Paz University Hospital and Principe de Asturias University Hospital).

**Methods:**

This is a quasi-experimental pre-post intervention study that examines two models of Mental Health Home Hospitalization as an alternative to full hospitalization. It will analyze various outcome variables, including clinical, functioning, satisfaction, quality of life, and costs /use of clinical resources.

**Results:**

Participants must be adolescents and young adults aged 12 to 18 years (inclusive), who present a psychiatric decompensation requiring an intensity of care similar to that provided in the hospital. Assessments, including CGI, HoNOSCA, C-SSRS, CSQ-8, EQ-5D-Y, will be conducted at the baseline visit, and at the end of the treatment. Resource use and costs will be recorded at the baseline and six months after the treatment.

**Conclusions:**

Home-based mental health hospitalization for adolescents is an emerging area of interest in the treatment of acute symptoms of mental disorders in this population, but there are not standard protocols for such interventions. Several issues remain to be clarified, including the care pathways for adolescents in crisis who require hospital care and whether organizational differences between interventions impact the outcomes.

**Disclosure of Interest:**

None Declared

